# Utilization and expenditures of veterans obtaining primary care in community clinics and VA medical centers: an observational cohort study

**DOI:** 10.1186/1472-6963-7-56

**Published:** 2007-04-18

**Authors:** Matthew L Maciejewski, Mark Perkins, Yu-Fang Li, Michael Chapko, John C Fortney, Chuan-Fen Liu

**Affiliations:** 1 Center for Health Services Research in Primary Care, Durham VA Medical Center, Department of Veterans Affairs, Durham, NC, USA; 2 Division of Pharmaceutical Outcomes and Policy, School of Pharmacy, University of North Carolina at Chapel Hill, Chapel Hill, NC, USA; 3 Health Services Research and Development, VA Puget Sound Health Care System, Department of Veterans Affairs, Seattle, WA, USA; 4 Department of Health Services, University of Washington, Seattle, WA, USA; 5 School of Nursing, University of Washington, Seattle, WA, USA; 6 Health Services Research and Development, Center for Mental Health and Outcomes Research, Central Arkansas Veterans Healthcare System, North Little Rock, AR, USA; 7 Division of Health Services Research, Department of Psychiatry, College of Medicine, University of Arkansas for Medical Sciences, Little Rock, AR, USA

## Abstract

**Background:**

To compare VA inpatient and outpatient utilization and expenditures of veterans seeking primary care in community-based outpatient clinics (CBOCs) and VA medical centers (VAMCs) in fiscal years 2000 (FY00) and 2001.

**Methods:**

The sample included 25,092 patients who obtained primary care exclusively from 108 CBOCs in FY00, 26,936 patients who obtained primary care exclusively from 72 affiliated VAMCs in FY00, and 11,450 "crossover" patients who obtained primary care in CBOCs and VAMCs in FY00. VA utilization and expenditure data were drawn from the VA's system-wide cost accounting system. Veteran demographic characteristics and a 1999 Diagnostic Cost Group risk score were obtained from VA administrative files. Outpatient utilization (primary care, specialty care, mental health, pharmacy, radiology and laboratory) and inpatient utilization were estimated using count data models and expenditures were estimated using one-part or two-part models. The second part of two-part models was estimated using generalized linear regressions.

**Results:**

CBOC patients had a slightly more primary care visits per year than VAMC patients (p < 0.0001), but lower primary care costs (-$71, p < 0.0001). CBOC patients had lower odds of one or more specialty, mental health, ancillary visits and hospital stays per year, and fewer numbers of visits and stays if they had any and lower specialty, mental health, ancillary and inpatient expenditures (all, p < 0.0001). As a result, CBOC patients had lower total outpatient and overall expenditures than VAMC patients (p < 0.0001).

**Conclusion:**

CBOCs provided veterans improved access to primary care and other services, but expenditures were contained because CBOC patients who sought health care had fewer visits and hospital stays than comparable VAMC patients. These results suggest a more complex pattern of health care utilization and expenditures by CBOC patients than has been found in prior studies. This study also illustrates that CBOCs continue to be a critical primary care and mental health access point for veterans.

## Background

The VA health care system serves 4.5 million veterans and has been establishing increasing numbers of Community-Based Outpatient Clinics (CBOCs) since 1995 to increase access to primary care for veterans [1]. CBOCs have been an essential component of the VA's transition from an inpatient care-oriented system of medical centers to an outpatient care-oriented organization that expanded primary care while containing costs. The VA has established two types of CBOCs – VA-staffed CBOCs and Contract CBOCs. VA-staffed CBOCs are clinics that the VA either owns or rents and are staffed by VA providers. Contract CBOCs are private practices staffed by non-VA providers that contract with the VA to provide primary care to veterans.

The VA made a major commitment to establishing CBOCs, which led to an evaluation of whether CBOC patients and veterans obtaining primary care at VA medical centers (VAMC) had comparable satisfaction, quality of care, utilization and expenditures. Satisfaction and quality of care were found to be comparable between CBOC and VAMC patients [2,3]. Utilization comparisons of veterans at 38 CBOCs and 32 VAMC primary care clinics showed that CBOC patients had more primary care encounters, fewer specialty care encounters, and similar hospital admission rates and length of stay as VAMC patients [4]. Of the 38 CBOCs in the Fortney (2002) study, 30 were VA-staffed CBOCs and 8 were Contract CBOCs. The 18 VA-staffed CBOCs included in the Maciejewski study [5] were a subset of the 30 CBOCs from the Fortney sample [4]. Comparisons of direct outpatient and inpatient expenditures of veterans at 18 VA-staffed CBOCs and 14 VAMCs showed that CBOC patients had lower specialty and total expenditures than VAMC patients, but similar primary care and inpatient expenditures [5].

In this paper, we extend the prior utilization and expenditure analyses of CBOC and VAMC patients in five ways. First, we examined total (indirect+direct) VA costs for CBOC, VAMC and crossover patients. The VA's cost accounting system has been more rigorously implemented and checked for data quality to enable inclusion of indirect costs that were excluded in the prior analyses. Second, we used a much larger sample of CBOCs (108 vs. 18), VAMCs (72 vs. 14) and veterans to strengthen the generalizability of our results. Third, we included data from fiscal years (FY) 2000 and 2001 to update the comparison. Fourth, we explicitly track "crossover" patients who obtained primary care at CBOCs and VAMCs in FY00 to assess whether this discontinuity in primary care is associated with different utilization and expenditures. In the prior study, these patients were lumped into the CBOC cohort and accounted for 12% of that group.

Fifth, we explicitly controlled for distance to directly examine how this factor affects health care use, to isolate the effects of CBOC provider practice patterns and organizational structure. The purpose of this analysis was to compare VA utilization and expenditures of veterans receiving primary care at CBOCs and VAMCs in FY00 and FY01. As the VA continues to rely upon CBOCs as a primary care delivery system that will improve access to care, it remains an open question as to whether CBOCs are actually fulfilling the stated goal of improving primary care access and containing costs.

## Methods

### Sample

To generate the sample for this study, we had to determine which CBOCs (and VAMCs to which they were affiliated) to include, and then which veterans from these facilities to sample. CBOCs were included based on the following criteria: 1) CBOCs had to have congressional approval, 2) the CBOC had to be open by 10/1/98 to ensure that cost data was tracked for three years to ensure stable estimates, 3) the clinic must have enrolled 200+ veterans in FY99 for sufficient power, 4) cost data had to be trackable separately from the affiliated VAMC. There were 744 CBOCs and other outpatient clinics in operation in FY00. Only 315 of these clinics were CBOCs that required Congressional approval, which was the original process for designating CBOCs. Another 429 outpatient clinics that have been retrospectively labeled CBOCs but did not require Congressional approval were excluded to ensure parity between the CBOCs examined in the prior study and this study. Of these 315 CBOCs, 180 were excluded because they did not meet the inclusion criteria of being in operation before 10/1/98, leaving 135 CBOCs. Twenty-two of the 135 CBOCs were excluded because they cared for fewer than 200 veterans in FY99. The 113 CBOCs with sufficient sample size was reduced by five because they couldn't be found in VA administrative databases (n = 3) or were closed in FY01 (n = 2), resulting in 108 CBOCs that ml the inclusion criteria mentioned above. These 108 CBOCs were affiliated with 72 VAMCs because several VAMCs had established multiple CBOCs. Seventy-six of these 108 CBOCs were VA-staffed CBOCs, and the remaining 32 were Contract CBOCs.

Veterans in this study were classified into one of three mutually exclusive groups based upon where the veteran obtained primary care in FY00: VAMC patients, CBOC patients or crossover patients. VAMC patients were defined as veterans who had no primary care visits to a CBOC and at least one primary care visit to a VAMC primary care clinic, including general internal medicine, geriatrics, women's health, or primary care. This definition of primary care stop codes was consistent with the primary care codes defined in previous studies [4,5]. VAMC patients could have no primary care visits to its affiliated CBOC, but could have visits to other unaffiliated CBOCs or VAMCs. CBOC patients were defined as veterans who had at least one visit to a CBOC and no primary care visits to the affiliated VAMC in FY00, but several CBOC patients had primary care visits at other VAMCs in FY00 not included in our sample. We defined them as CBOC patients for the purpose of this study because veterans may seek care at VAMCs not affiliated with their CBOC during vacations or other reasons. A crossover patient was a veteran who had at least one visit to a CBOC and an affiliated VAMC primary care clinic in FY00. In the prior CBOC analyses, 12% of CBOC patients had VAMC and CBOC primary care but were included in the CBOC group [4]. Crossover patients were separately categorized from CBOC patients to examine whether their utilization and expenditures were different from VAMC and CBOC patients' use. We contrast the experience of CBOC patients as a group against the experience of VAMC patients and crossover patients in this paper, and examine the utilization and expenditure differences between Contract and VA-staffed CBOC patients in a separate paper (available from authors).

A random sample of 250 patients was drawn from each CBOC and VAMC primary care population to obtain sufficient power for all analyses and frequency weights were computed for use in regression analysis. For CBOCs with 200–250 enrolled veterans, all veterans were drawn for the study sample and frequency weights for regression analysis equalled 1.0. All crossover patients receiving primary care in the CBOCs and affiliated VAMCs in our sample were included and were assigned frequency weights of 1.0. Patients were then excluded if they didn't survive until the end of FY01 (n = 6,754), or had missing (n = 74) or excessive (e.g., >200 miles) travel distance from their home to the closest VAMC (n = 4,688), which resulted in 23,894 patients in 108 CBOCs, 26,176 patients from the 72 affiliated VAMCs, and 11,074 crossover patients.

### Data

The five datasets used in this study were drawn from administrative files that are maintained at the VA's central data repository, the Austin Automation Center. The first two datasets – the FY00 Outpatient Care File (OPC) and FY00 Patient Treatment File (PTF) – contained demographic information, county of residence, and utilization data that were used to identify the study sample and control for covariates related to health care utilization and expenditures. The outpatient and inpatient utilization files report the location of care (CBOC or VAMC), clinic stop codes that were used to identify the type of care, clinic visit date or admission and discharge dates, and diagnosis and procedure codes.

The second two datasets – the FY00 and FY01 Decision Support System (DSS) Outpatient and Inpatient National Extracts – contained similar utilization and diagnostic data as OPC and PTF, but also have direct, indirect and total expenditures for each VA outpatient visit and inpatient hospitalization. Expenditures were inflation-adjusted using the Medical Component of the Current Price Index. We also obtained 1999 Diagnostic Cost Groups (DCG) risk scores that are routinely generated for all veterans receiving care in a given year to adjust for differential risk between patient groups, because DCG was predictive of utilization and expenditures in the prior CBOC studies [4,5].

The dependent variables for our analysis included outpatient and inpatient utilization and expenditure variables. Outpatient utilization and expenditures were partitioned into different types of care by clinic stop codes (e.g., primary care, mental health, specialty, pharmacy, radiology, laboratory, other) and summarized into total outpatient visits and expenditures. In the VA, lab space is physically separated from provider clinics, so veterans have to go to VA laboratories to have one or more tests done in a single lab "visit". Radiology and pharmacy "visits" work in the same way.

Inpatient utilization was defined as the number of hospitalizations, and inpatient expenditures were defined as the sum of expenditures across all hospitalizations in the same year. Demographic information used as independent variables regression analysis included age, gender, race, marital status, means test-based eligibility for free care, service-related disability, and 1999 DCG risk score. We also adjusted for distance between veteran's zipcode and the veteran's usual source of care (e.g., VAMC or CBOC) to control for access issues related to VAMC and CBOC travel costs. Differential distance, defined as (Distance between VAMC county and home county of residence) – (Distance between CBOC county and home county of residence), was used in primary care analyses, but distance between VAMC county and home county of residence was used to analyze all other outcomes.

### Analysis

To assess whether CBOCs influence the probability of care and/or the level of care, we estimate three measures of VA resource use – 1) odds of service use, 2) number of outpatient visits or inpatient hospitalizations, and 3) expenditures incurred. Ninety percent of veterans had primary care and outpatient pharmacy visits and 95% of veterans had one or more outpatient visits of all kinds, so the odds of use were not estimated for these two outcomes. Bivariate comparisons of odds of service were assessed via analysis of variance methods and bivariate comparisons of utilization and costs were assessed by the Cuzick extension of the nonparametric Wilcoxon rank-sum test for three or more groups [6].

In multivariate analysis, one-part models and two-part models of utilization and costs were run. One-part models were run for outcomes that had few observations with zero use (e.g., primary care, total outpatient, and overall care). Two-part models are used to estimate the odds of use and the level of use separately for those that use health care [7]. A hurdle variant of the two-part model was run for outcomes that had many observations with zero values (e.g., mental health, specialty, radiology, laboratory, other outpatient, inpatient) because of the poor numerical properties of count data models (e.g., zero-inflated negative binomial models) that use the same covariates in both parts of the model [8]. The odds of mental health, specialty, and ancillary (radiology, laboratory, and other) outpatient service use, as well as odds of inpatient admission, were estimated using logit regressions. The number of outpatient visits and number of hospitalizations were estimated using count data methods [8]. The number of mental health, specialty, and ancillary visits for those with visits, and the number of hospitalizations for those who were hospitalized, was estimated with negative binomial models. This "modified" two-part model approach allows the conventional, separate modeling of the process for generating zeros and a count data model for users that explicitly accounts for overdispersion. Poisson regression models were estimated for total outpatient visits, primary care visits, and outpatient pharmacy visits because overdispersion due to high utilizers was not a problem [9].

Expenditure models for overall care, outpatient care, primary care and outpatient pharmacy were estimated using one-part generalized linear models (GLMs) with a normal distribution and log link function to restore log-normality. GLMs have been shown to fit expenditures with significant heteroskedasticity better than ordinary least squares [10] and have the added benefit of yielding predicted expenditures without having to retransform log costs. In a comparison of various generalized linear models and ordinary least squares from another CBOC study [11], the GLM with normal distribution and log link fit the middle three quintiles of the expenditure distribution better than OLS, based on predictive ratios [12]. Inpatient, specialty, mental health, radiology, laboratory or other outpatient expenditures for those with one or more visits were also estimated using GLMs with a normal distribution and log link function. Specifications, cluster corrections for repeated measures on each veteran, and sampling weights were standardized across all regressions. Significance was lowered from 0.05 to 0.01 to adjust for multiple comparisons.

Since veterans were not randomly assigned to groups, we used propensity score matching with quintiles to improve the balance in the observed characteristics of veterans seen in CBOCs and VAMCs [13]. To account for the possibility that the clustering of CBOCs within VAMCs and clustering of VAMCs within Veteran Integrated Service Networks (VISNs) might impact our variance estimates, we also tested models that included VAMCs as random effects and VISNs as fixed effects. These propensity score and cluster adjustments obtained similar results to our unadjusted models, so the unadjusted models are presented.

## Results

### Patient characteristics

CBOC patients are younger and more likely to be married than VAMC and crossover patients (Table 1). Two proxies of health risk – veterans' service-related disability and DCG risk score – are lower for CBOC patients than VAMC or crossover patients. A lower percentage of VAMC patients are above the means-test threshold and required to pay VA copayments than CBOC and crossover patients. Finally, CBOC patients live farther from VAMCs than VAMC and crossover patients, which results in greater differential distances (Table 1).

**Table 1:** Demographic Characteristics of CBOC, VAMC and Crossover Patients

	*VAMC Patients*	*CBOC Patients*	*Crossover Patients*
Age	60.4 (14.3)	64.0 (13.0)	60.0 (14.2)
Male (%)	94.6	96.4	92.3
Married (%)	54.1	67.8	58.9
Not Married (%)	43.7	31.7	39.8
Unknown Marital (%)	2.2	1.5	1.3
Caucasian (%)	51.3	48.0	60.2
Non-Caucasian (%)	17.3	6.8	12.1
Unknown Race (%)	31.4	45.2	27.7
Service Connection (%)	15.9 (28.0)	11.5 (23.6)	21.6 (30.9)
DCG score, 1999	0.61 (1.00)	0.34 (0.64)	0.62 (0.98)
Means test status (%)			
No means test required	36.7	30.4	46.4
Above means test	15.9	27.0	22.2
Below means test	44.7	41.8	39.9
Not applicable	2.7	0.8	1.5
Differential Distance	5.7 (33.0)	44.5 (35.6)	34.8 (37.1)
Home to VAMC Distance	27.5 (31.0)	60.7 (37.7)	51.8 (39.0)
Sample Size	26,176	23,894	11,074

### Unadjusted utilization and cost

The odds of primary care, pharmacy and total outpatient use were significantly different between CBOC, VAMC and crossover patients, but the differences were not large (Table 2, columns 2–4). Bigger differences in the odds of use were found in specialty, mental health, ancillary care, and for inpatient care. CBOC patients had significantly lower odds of use of these services than VAMC patients, who had significantly lower odds of use than crossover patients (p < 0.001). The average number of outpatient visits and inpatient admissions per person per year were also significantly lower for CBOC patients than for VAMC patients (p < 0.001). CBOC patients had 3.3 primary care visits per year on average, while VAMC patients had 3.6 primary care visits per year and crossover patients had 5.0 primary care visits per year (p < 0.001). The same trend was found in VAMC-based specialty care visits per year (2.1 for CBOC patients vs. 4.9 for VAMC and crossover patients, p < 0.001), and total outpatient visits per year (23 visits for CBOC patients vs. 36 visits for VAMC patients vs. 40 visits for crossover patients, p < 0.001) (Table 2, columns 5–7).

**Table 2:** Unadjusted Utilization and Expenditures of CBOC, VAMC and Crossover Patients in 2000–2001

	*Patients with at least one Visit or Admission*			*Number of visits or admissions Per Person Per Year*			*Expenditures Per Patient Per Year*		
	CBOC	VAMC	Crossover	CBOC	VAMC	Crossover	CBOC	VAMC	Crossover
Outpatient									
Primary Care	94%	93%	96%	3.3 (2.8)	3.6 (3.1)	5.0 (3.9)	$381 (438)	$509 (626)	$649 (720)
Specialty Care	48	72	75	2.1 (4.0)	4.9 (8.0)	4.9 (6.5)	413 (1706)	1013 (3568)	1077 (4098)
Mental Health	13	23	27	0.8 (4.9)	2.8 (14)	2.6 (12)	102 (665)	342 (1733)	302 (1363)
Pharmacy	90	90	95	--	--	--	684 (2009)	936 (2367)	1062 (2083)
Radiology	30	55	58	0.6 (1.2)	1.3 (1.9)	1.4 (2.0)	111 (717)	251 (524)	287 (556)
Laboratory	68	83	83	2.2 (2.9)	3.5 (4.6)	3.7 (4.3)	119 (332)	208 (410)	238 (3473)
Total Outpatient	97	97	99	23 (22)	36 (38)	40 (34)	2092 (3692)	3921 (6294)	4258 (7235)
Inpatient	5	13	15	0.1 (0.4)	0.2 (0.8)	0.2 (0.8)	656 (5120)	1867 (9306)	1964 (9042)
OVERALL	--	--	--	--	--	--	2748 (6936)	5788 (12351)	6222 (12701)
Sample Size	23,894	26,176	11,074	23,894	26,176	11,074	23,894	26,176	11,074

All outcomes are significantly different at a p-value < 0.0001;

Numbers in parentheses refer to Standard Deviations;

All analyses were adjusted for sampling weights.

Unadjusted VA outpatient expenditures followed the patterns of outpatient utilization (Table 2, columns 8–10). Average primary care expenditures per person per year were lowest for CBOC patients and highest for crossover patients ($381 for CBOC, $590 for VAMC and $649 for crossover patients, p < 0.001). Total outpatient expenditures averaged $2,092 for CBOC patients, $3,921 for VAMC patients, and $4,258 for crossover patients (p < 0.001). Average inpatient expenditures per year averaged $656 for CBOC patients, $1,867 for VAMC patients and $1,964 for crossover patients (p < 0.001). Overall expenditures averaged $2,748 for CBOC patients, $5,788 for VAMC patients, and $6,222 for crossover patients (p < 0.001).

### Adjusted utilization and cost

Odds ratios and predicted visit and cost differences adjusted for age, gender, race, marital status, service connection, distance, means-test status, and DCG risk are listed in Table 3. CBOC patients had 0.1 fewer adjusted primary care visits per year as VAMC patients, and had lower primary care expenditures (-$81, both p < 0.0001). CBOC patients had significantly lower odds of having specialty, mental health, and ancillary (radiology, laboratory and other outpatient) visits than VAMC patients (p < 0.0001 for all). CBOC patients with use had fewer specialty, mental health, outpatient pharmacy, and ancillary visits than VAMC patients that used these services. Lower CBOC utilization (for users) translated into significantly lower expenditures for these same services, which resulted in lower total outpatient expenditures compared to VAMC patients (-$923, p < 0.0001). CBOC patients were less likely to be hospitalized than VAMC patients (odds = 0.48, p < 0.0001), but those CBOC patients who were hospitalized had similar expenditures as VAMC patients who were hospitalized. As a result of significantly lower total outpatient expenditures, CBOC patients had lower overall expenditures (-$1551, p < 0.0001) per person per year than VAMC patients.

**Table 3:** Adjusted Utilization and Expenditures of CBOC, VAMC and Crossover Patients in 2000–2001

	*Odds Ratio (for two-part models only)*		*Difference in predicted Visits or admissions per patient per year*		*Difference in Predicted Expenditures Per Patient Per Year*	
	CBOC	Crossover	CBOC	Crossover	CBOC	Crossover
Outpatient						
Primary Care^a^	--	--	0.10 (0.03)***	1.34 (0.04)***	-71 (5)***	130 (6)***
Specialty Care^b^	0.46 (0.01)***	1.10 (0.03)	-1.42 (0.07)***	-0.10 (0.08)	-339 (33)***	60 (47)
Mental Health^b^	0.78 (0.02)***	1.14 (0.03)***	-1.85 (0.35)***	-0.60 (0.35).	-282 (43)***	-78 (47).
Pharmacy^a^	--	--	--	--	-81 (20)***	103 (17)***
Radiology^b^	0.41 (0.01)***	1.05 (0.02)*	-0.39 (0.02)***	0.03 (0.03)	-58 (14)***	34 (8)***
Laboratory^b^	0.45 (0.01)***	0.90 (0.03)***	-0.41 (0.04)***	0.31 (0.04)***	-34 (4)***	29 (16).
Total Outpatient^a^	--	--	--	--	-923 (39)***	364 (52)***
Inpatient^b^	0.48 (0.02)***	1.08 (0.03)	-0.12 (0.04)***	0.01 (0.03)	-109 (577)	81 (497)
OVERALL^a^	--	--	--	--	-1551 (70)***	491 (91)***
Sample Size	126,956		Variable		Variable	

Reference Group is VAMC patients; ***P-value < 0.0001, **P-value < 0.001, *P-value < 0.01;

Utilization and expenditure regressions control for age, age-squared, female gender, age and gender interaction, marital status, race, veteran percent service connection, distance, DCG risk score, copay status and time (2000);

All analyses were adjusted for sampling weights;

a – one-part models of utilization and costs estimated on the entire sample and odds of use was not estimated

b – two-part models of utilization and costs estimated on veterans with use > 0 and odds of use estimated on entire sample

Crossover patients who obtained primary care in CBOCs and VAMCs in FY00 had 1.3 more primary care visits and higher primary care expenditures than VAMC patients in both years (p < 0.0001). They also had greater odds of mental health and radiology visits, but lower odds of laboratory visits. Crossover patients with use had more outpatient pharmacy visits and expenditures ($103, p < 0.0001) than VAMC patients. Total outpatient expenditures and overall expenditures were significantly higher for crossover patients ($364 and $491, respectively).

Distance was a significant determinant of primary care utilization and expenditures, as well as radiology, laboratory and outpatient pharmacy utilization (p < 0.0001, not reported). Greater travel distance to a VAMC was associated with higher odds of being hospitalized (p < 0.01), but did not affect the level of inpatient expenditures once a veteran was hospitalized. This result is inconsistent with an earlier study that found that distance is positively associated with inpatient utilization [14].

## Discussion

CBOCs have been an integral part of the VA's continuing transition into a primary care-focused health care system. These outpatient clinics were established to improve veteran access to primary care and contain total health care expenditures, and they appear to be providing primary care access without a commensurate increase in primary care expenditures. Overall expenditures of CBOC patients were $1,588 lower than for VAMC patients because of lower use of inpatient, specialty, mental health and outpatient ancillary care services provided at VAMCs for those veterans who received one or more services, despite the fact that the odds of using these services was higher for CBOC patients. These utilization and expenditure differences held even when relative distance, demographic characteristics and patient risk were controlled [15,16].

The results from this study differ from earlier studies that used smaller samples in 1998. CBOC-VAMC visit differences were smaller in this study than in an earlier study [4] for primary care (0.1 visits vs. 1.7 visits in prior study) and specialty care (-0.4 visits vs. -1.8 in prior study), but hospital admissions were similar. CBOC-VAMC expenditure differences were similar for specialty care and inpatient care, but were significantly different for primary care (-$71 vs. $30 in prior study) [5]. The differences in total costs from this study were five times larger than that from the earlier study (-$1551 vs. -247 in prior study). These expenditure differences could be due to a number of factors, including a larger sample size (108 vs. 18), the inclusion of Contract CBOCs into this sample, and more complete adjustment for confounders such as distance. Distance to a VAMC was an important determinant of utilization and expenditures, particularly for VAMC-based services, and this variable was not controlled in the earlier studies. Time and travel costs incurred for accessing CBOCs and VAMCs might have been an important factor contributing to lower specialty visits and expenditures of CBOC patients, because distance has shown to be an important determinant of veterans' services use in other studies [17-19].

This study had several limitations that must be acknowledged. First, veterans were not randomized to VA treatment site, so it is possible that CBOCs experienced favorable selection and unmeasured covariates (e.g., disease severity, propensity to seek primary care) related to CBOC status and resource use could have biased the effect of CBOC on utilization and expenditures [20]. We attempted to reduce unobserved confounding related to CBOC status and resource use by controlling for demographic characteristics and patient risk (via the DCG risk score), distance, copayment status, marital status and service-related disability, but this confounding may not have been completely eliminated. Second, a consistent specification was used across all outcomes to simplify presentation, which may have led to specification biases that would modify our findings. Third, we had no data on health outcomes and only limited data on quality of care that showed no difference in provision of diabetic eye exams and flu shots for patients with COPD. In the absence of more complete quality of care and health outcome measures, it is unknown whether lower utilization and expenditures had adverse effects for veterans at CBOCs. Fourth, our CBOC sample included early entrants that had been in operation for three years by 2001, so our results may not generalize to CBOCs that have been established more recently or to the start-up phase of CBOCs. Finally, we did not have data on non-VA utilization (e.g., Medicare) to assess whether VA differences were offset by non-VA utilization and cost differences. These results represent these veterans' VA experience only.

As the current veteran population ages and develops more chronic conditions and veterans from the Iraq Theater enter the VA system, it will be useful to examine whether relatively healthier veterans will continue to choose CBOC care or if the health profile of CBOC and VAMC patients become more similar. If the health profile of patients becomes more similar over time and the care of CBOC patients becomes more complex, CBOC-VAMC cost differences observed in this study may change. CBOCs will be challenged to maintain their current performance, because CBOCs may have created strong patient-provider relationships that patients valued enough to seek CBOC care in situations where specialty care would have been appropriate. CBOC patients do not have onsite access to the broad range of specialty and mental health clinics available on VAMC campuses. CBOC providers may also have differed from VAMC providers in conformity with evidence-based medicine [21].

## Conclusion

CBOCs provided veterans improved access to primary care and other services, but expenditures were contained because CBOC patients who sought health care had fewer visits and hospital stays than comparable VAMC patients. These results suggest a more complex pattern of health care utilization and expenditures by CBOC patients than has been found in prior studies, but also illustrates that CBOCs continue to be a critical primary care and mental health access point for veterans.

It will be important to track CBOC performance into the future as the program matures and CBOCs add services, such as the recently mandated mental health capacity. Future investigations should examine what types of CBOC patients return to VAMCs for all of their health care needs, how CBOC and VAMC primary care clinic staffing, practice styles, management and decision technology differ, what factors are associated with CBOCs being primary care centers of excellence, and how the new Medicare prescription drug benefit affects elderly veterans' use of CBOCs and VAMC primary care clinics. CBOCs are currently in operation throughout the VA health care system and appear to be serving the VA and veterans as intended.

## Competing interests

All of the authors are employees of and receive salary from the Health Services Research and Development service of the Veterans Health Administration, which administers CBOC and VAMC services to veterans. Otherwise, we have no financial or non-financial competing interests.

## Authors' contributions

MLM, CFL, MC and JCF conceived of the study, wrote both grant applications, designed the analyses, interpreted the results, and drafted and revised the manuscript. YFL and MP cleaned and linked all of the data files, and conducted all of the statistical analyses. All authors read and approved the final manuscript.

## Pre-publication history

The pre-publication history for this paper can be accessed here:


